# 城市居民有机磷酸酯内暴露特征及健康风险评估

**DOI:** 10.3724/SP.J.1123.2025.01006

**Published:** 2025-06-08

**Authors:** Huawei WANG, Shiyu SHI, Ling LIU, Ding CHEN, Zhixian LYU, Ziyi SONG, Youjie WANG, Lulu SONG, Surong MEI

**Affiliations:** 1.武汉市洪山区疾病预防控制中心，湖北 武汉 430070; 1. Hongshan District Center for Disease Control and Prevention，Wuhan 430070，China; 2.华中科技大学公共卫生学院环境医学研究所，教育部环境与健康重点实验室，湖北 武汉 430030; 2. Key Laboratory of Environment & Health of Ministry of Education，Institute of Environmental Medicine，School of Public Health，Huazhong University of Science and Technology，Wuhan 430030，China; 3.华中科技大学同济医学院公共卫生学院 儿少卫生与妇幼保健学系，湖北 武汉 430030; 3. Department of Maternal and Child Health，School of Public Health，Tongji Medical College，Huazhong University of Science and Technology，Wuhan 430030，China

**Keywords:** 有机磷酸酯, 尿液, 影响因素, 暴露特征, 健康风险评估, organophosphate esters （OPEs）, urine, influencing factors, exposure profiles, health risk assessment

## Abstract

本研究以我国城市地区1 869名普通成人为研究对象，采用超高液相色谱-串联质谱技术（UPLC-MS/MS）测定人体尿液样品中15种有机磷酸酯（OPEs）代谢物的含量水平，探讨了性别、年龄、体质指数（BMI）、是否吸烟、运动频率、家庭收入，以及各类食品摄入对尿液中OPEs代谢物含量水平的影响，并进一步根据尿液中OPEs代谢物的含量水平评估OPEs的每日摄入量（EDI），结合非致癌风险参考剂量（RfD）计算相应的潜在非致癌风险，以风险熵（HQ）和危害指数（HI）来表示OPEs各单体和总体累积暴露的健康风险。结果表明，6种OPEs代谢物在人体尿液中的检出率大于60%，以磷酸二丁氧乙酯（BBOEP）和1-羟基-2-丙基（1-氯-2-丙基）磷酸酯（BCIPHIPP）为主，含量分别为0.56 ng/mL和0.36 ng/mL。男性尿液中磷酸二（1，3-二氯-2-丙基）酯（BDCIPP）、BCIPHIPP和BBOEP的含量水平高于女性，而女性尿液中4-羟基苯基-磷酸苯基酯（4-HO-DPHP）的含量高于男性。BCIPHIPP和磷酸二苯酯（DPHP）的含量水平与年龄呈负相关关系，而BCIPHIPP和磷酸邻二苯甲酯（DoCP）/磷酸对二苯甲酯（DpCP）的含量水平与家庭收入表现出正相关关系。较高的运动频率有效降低了尿液中BDCIPP和BCIPHIPP的含量水平。研究人群OPEs的EDI值为104 ng/（kg⋅d） bw，且男性高于女性，磷酸三丁氧乙酯（TBOEP）对于EDI的贡献最大，占总EDI值的55.6%。本研究中绝大多数研究对象的HI值小于1，表明不存在明显的非致癌风险，但整体而言男性的HI值高于女性，且TBOEP是主要的高风险单体，贡献了总HI值的68.9%。综上所述，本研究人群普遍暴露于OPEs，且男性具有更高的暴露水平和健康风险，表明OPEs暴露存在性别差异。本研究揭示了我国城市居民OPEs的暴露水平及特征，为后续研究及防控政策制定提供了数据支持和科学依据。

## Abstract (en)

The contents of 15 organophosphate ester （OPE） metabolites in the urine of 1 869 adults residing in urban areas were quantified using ultra performance liquid chromatography-tandem mass spectrometry （UPLC-MS/MS）. How gender， age， body mass index （BMI）， smoking status， exercise frequency， family income and dietary intake affected the contents of OPE metabolites in human urine were discussed. Furthermore， the daily intake （EDI） of OPEs was evaluated based on the contents of OPE metabolites in urine. The corresponding potential non-carcinogenic risks were calculated in combination with the non-carcinogenic risk reference dose （RfD）， with the health risks of individual OPE monomers and overall cumulative exposure expressed using the hazard quotient （HQ） and hazard index （HI）. Six OPE metabolites exhibited detection frequencies in excess of 60%， with bis（2-butoxyethyl） phosphate （BBOEP） and 1-hydroxy-2-propyl bis（1-chloro-2-propyl） phosphate （BCIPHIPP）， as the two main OPE metabolites， detected at levels of 0.56 and 0.36 ng/mL， respectively. Men exhibited higher urine contents of bis（1，3-dichloro-2-propyl） phosphate （BDCIPP）， BCIPHIPP， and BBOEP than women， whereas women exhibited higher urine contents of 4-hydroxyphenyl-phenylphosphate （4-HO-DPHP）. The levels of BCIPHIPP and diphenyl phosphate （DPHP） were found to correlate negatively with age， while the BCIPHIPP， and di-*o*-tolyl-phosphate （DoCP）/di-*p*-tolyl-phosphate （DpCP） levels correlated positively with family income. Higher exercise frequencies were found to be associated with significantly lower levels of BDCIPP and BCIPHIPP in urine. Furthermore， the frequency of nut consumption and the level of 4-HO-DPHP in urine were determined to be significantly negatively correlated. This study did not identify any significant associations between contents of urinary OPE metabolites and smoking or the intake of other foods， which suggests that smoking and dietary intake are not the primary OPE exposure pathways for the investigated population. Future research should have broader scope to elucidate the principal OPE exposure pathways. The overall OPE exposure levels for all participants in this study ranged between 5.60 and 2 800 ng/（kg⋅d） bw， with a median exposure level of 104 ng/（kg⋅d） bw. Among the four OPE monomers， Tris（2-butoxyethyl） phosphate （TBOEP） exhibited the highest exposure level， with a median value of 57.2 ng/（kg⋅d） bw （ranging between 1.11 and 1 330 ng/（kg⋅d） bw）， thereby contributing up to 55.6% of the total OPE exposure. Additionally， tri-*n*-butyl phosphate （TNBP） also exhibited significant exposure， with a median level of 32.4 ng/（kg⋅d） bw （ranging between 0.138 and 2 000 ng/（kg⋅d） bw）， which accounts for 31.5% of the total OPE exposure. Gender-based analysis revealed that men exhibited higher OPE exposure levels than women. Specifically， men exhibited a median exposure level of 112 ng/（kg⋅d） bw （ranging between 6.03 and 2 670 ng/（kg⋅d） bw） compared to the value of 89.9 ng/（kg⋅d） bw （ranging between 5.61 and 2 800 ng/（kg⋅d） bw） recorded for women. The vast majority of study participants exhibited HI values of less than one， indicative of no obvious non-carcinogenic risks. The OPEs exhibited HI values in the 0.000 2–1.03， with a median value of 0.06. The exposure risks associated with the four OPE monomers are ranked in following order： TBOEP （median HI=0.038， range： 0.000 7–0.883）， TNBP （median HI=0.013， range： 0.000 05–0.833）， tris（1，3-dichloro-2-propyl） phosphate （TDCIPP） （median HI=0.002， range： 0.000 8–0.288）， and triphenyl phosphate （TPHP） （median HI=0.001， range： 0.000 2–0.350）； these monomers contribute 68.9， 24.4， 4.2， and 2.5% to the overall HI value， respectively. Among all study participants， men exhibited a higher exposure risk （median HI=0.061， range： 0.002–1.03） than women （median HI=0.049， range： 0.002–0.840）. Notably， TBOEP was identified as the primary high-risk monomer for both genders， contributing 70.1% （median HI=0.042， range： 0.000 7–0.883） and 67.6% （median HI=0.035， range： 0.000 8–0.835） to the overall health risks of men and women， respectively. In conclusion， the study population was ubiquitously exposed to OPEs， with men exhibiting higher exposure levels and associated health risks， which suggests that OPE exposure levels are gender-dependent. This study revealed the exposure levels and profiles of OPEs of urban residents， and provides supporting data and a scientific foundation for subsequent studies and policy formulations.

有机磷酸酯（OPEs）是一类含有三酯结构的卤化和非卤化有机化合物，随着多溴二苯醚（PBDEs）和六溴环十二烷（HBCDs）等传统有机阻燃剂的全面禁用，其作为替代品被广泛用于各类生产生活商品中^［1‒3］^。近年来，由于在各类环境介质中频繁检出，OPEs受到了广泛关注^［4‒7］^。有研究指出，OPEs主要通过灰尘摄食、呼吸吸入及皮肤吸收等途径从环境进入人体，并在人体各个组织和器官中蓄积、代谢和清除^［8‒11］^。此外，流行病学和毒理学研究表明，长期暴露于OPEs可能导致包括内分泌干扰、不良出生结局和生殖毒性在内的一系列负面健康效应^［12‒15］^。因此，阐明OPEs的人体暴露水平并评估其健康风险对提升人类健康生活水平至关重要。

然而，现有的研究仍然存在诸多分歧，不同外暴露模型的OPEs生物有效性参数并不一致、相应的皮肤渗透系数缺乏统一标准以及呼吸暴露未考虑空气颗粒物的吸入有效性等，导致尚无法通过外暴露方法准确评估OPEs的暴露剂量以及准确识别其主要暴露路径^［4］^，因而给OPEs暴露的评估带来了诸多困扰。鉴于此，研究人员近年来尝试使用内暴露评估方法来探究OPEs的人体暴露情况^［16‒18］^。尿液作为一种非侵入性基质，通常能够反映短期暴露情况，已被认定为体内接触OPEs的有效指标。相比于外暴露评估方法，内暴露监测能够提供一定时期内人体通过各种途径暴露于OPEs的整体情况，从而避免由外部暴露评估引起的误差^［19］^。因此，对OPEs代谢物的内暴露水平进行研究对于进一步揭示OPEs暴露的健康风险尤为重要。

OPEs的内暴露水平受到多种因素的影响，包括人口学特征、生活方式和饮食习惯等，这些因素可能进一步影响其健康风险水平^［20］^。因此，在评估OPEs的健康风险时，采用内暴露方法需要深入研究不同性别、生活方式和饮食习惯的差异。本研究选取我国城市居民普通人群作为研究对象，采用超高效液相色谱-串联质谱（UPLC-MS/MS）技术测定尿液中15种OPEs代谢物的含量水平，以评估其相应的潜在非致癌风险。该研究旨在阐明OPEs的暴露水平及其相关影响因素，并进一步评估单一和累积暴露的健康风险。

## 1 实验部分

### 1.1 仪器、试剂与材料

超高效液相色谱-串联质谱仪（日本Shimadzu公司），隔膜真空泵（美国GAST公司），氮气发生器（武汉科森仪器有限公司），氮吹仪（上海安谱实验科技股份有限公司），涡旋振荡仪（MS200，杭州瑞诚仪器有限公司）。二氯甲烷、丙酮（色谱纯，美国Fisher公司），甲醇、乙腈、正己烷、甲基叔丁基醚（色谱纯，德国Merck公司），*β*-葡萄糖醛酸酶（500 000 U，德国Merck公司），乙酸钠（分析纯，国药集团有限公司），纯净水（杭州娃哈哈集团有限公司）。

OPEs代谢物标准品：磷酸二丁氧乙酯（BBOEP）、磷酸二（1-氯-2-丙基）酯（BCIPP）、磷酸三（2-氯乙基）酯（TCEP）、磷酸二（1，3-二氯-2-丙基）酯（BDCIPP）、磷酸二正丁酯（DNBP）、磷酸二苯酯（DPHP）、和磷酸邻二苯甲酯（DoCP）/磷酸对二苯甲酯（DpCP）标准品购自Toronto Research Chemicals公司（加拿大），1-羟基-2-丙基（1-氯-2-丙基）磷酸酯（BCIPHIPP）、3-羟基苯基-磷酸二苯酯（3-HO-TPHP）、4-羟基苯基-磷酸二苯酯（4-HO-TPHP）、4-羟基苯基-磷酸苯基酯（4-HO-DPHP）、双（2-丁氧基乙基）3′-羟基-2-丁氧基乙基磷酸酯（HO-TBOEP）、2-乙基-5-羟基己基二苯基磷酸酯（5-HO-EHDPP）、2-羟基二丁氧基磷酸酯（BBOEHEP）和2-乙基己基苯基磷酸酯（EHPHP）标准品由Adrian Covaci实验室（比利时）提供^［21］^。

同位素内标：磷酸三（2-氯乙基）酯-D_12_（TCEP-D_12_）、磷酸二（1，3-二氯-2-丙基）酯-D_10_（BDCPP-D_10_）、磷酸二苯酯-D_10_（DPHP-D_10_）、磷酸二丁氧乙酯-D_4_（BBOEP-D_4_）、2-羟基二丁氧基磷酸酯-D_4_（BBOEHEP-D_4_）和磷酸三丁氧乙酯-D_6_（TBOEP-D_6_）购自Toronto Research Chemicals公司（加拿大），磷酸三苯酯-D_15_（TPHP-D_15_）购自Wellington公司（美国）。

### 1.2 标准溶液配制

将OPEs代谢物标准品溶于甲醇溶液，配制质量浓度均为20 μg/mL的15种OPEs混合标准溶液。之后将混合标准溶液用甲醇依次稀释，配制质量浓度为0.1、0.2、0.5、1、2、5、10、20和50 ng/mL的系列混合标准溶液。OPEs代谢物的同位素内标同样使用甲醇溶液溶解，配制质量浓度均为1 ng/mL的8种内标混合标准溶液。上述所有标准溶液均储存于‒20 ℃的冰箱中备用。

### 1.3 尿液样品采集

本研究从我国中部地区某三甲医院健康管理中心随机招募成年人作为研究对象，并在随访期间采用无菌聚丙烯离心管收集参与者的尿液样本，在低温条件下运送至实验室‒20 ℃冰箱中保存。此外，对采样人员进行了包括性别、年龄、教育程度、身高、体重、体质指数（BMI）、吸烟和体育锻炼资料等信息的问卷调查。排除样本和问卷信息缺失者，最终共有1 869名参与者被纳入此项研究。本研究方案已通过华中科技大学同济医学院伦理委员会的批准（伦理文件批号：［2018］IEC（S329）号）。所有志愿者均自愿参加，并签署知情同意书。

### 1.4 样品前处理

尿液样品以3 000 r/min离心10 min后取1 mL上清液于洁净离心管中，加入200 μL 1 ng/mL的内标混合标准溶液，然后加入1 000 U *β*-葡糖糖醛酸酶和350 μL乙酸钠缓冲液（pH=5），涡旋均匀后，在37 ℃水浴下孵育12 h。进一步在孵育完毕的样品中加入3 mL乙腈，涡旋0.5 min，以8 000 r/min离心3 min。之后加入5 mL甲基叔丁基醚，以8 000 r/min离心3 min后，从上层分离约7.5 mL有机相。将有机相使用氮吹仪蒸发至近干并使用100 μL甲醇-水（1∶9，v/v）混合溶液定容，在通过0.22 μm滤膜后转移至进样瓶中待分析。

### 1.5 仪器分析

采用Agilent Zorbax Eclipse Plus C18色谱柱（50 mm×2.1 mm，1.8 μm），色谱柱温度30 ℃，进样体积4 μL，流速0.2 mL/min。采用电喷雾离子源（ESI）和多反应监测模式（MRM）测定尿液样本中的OPEs代谢物。4-HO-DPHP、BCIPP、BCIPHIPP、DNBP、BDCIPP、DPHP、BBOEP、DoCP/DpCP、3-HO-TPHP、4-HO-TPHP和EHPHP在ESI^‒^模式下测定，以5.00 mmol/L醋酸铵水溶液和5.00 mmol/L醋酸铵甲醇溶液分别作为流动相A和流动相B。其他化合物在ESI^+^模式下测定，流动相C和D分别为超纯水和甲醇。流动相梯度程序如表1所示，各单体的质谱参数见表2。

**表 1:** 梯度洗脱程序

ESI^‒^			ESI^+^		
Time/min	*φ*（A）/%	*φ*（B）/%	Time/min	*φ*（C）/%	*φ*（D）/%
0	90	10	0	70	30
2.0	75	25	2.0	25	75
5.0	75	25	5.0	20	80
5.5	50	50	5.5	10	90
7.5	50	50	7.5	5	95
9.0	5	95	9.0	5	95
14.0	5	95	11.0	0	100
14.5	90	10	16.0	0	100
17.0	90	10	17.0	70	30
			20.0	70	30

A： 5.00 mmol/L ammonium acetate in water； B： 5.00 mmol/L ammonium acetate in methanol； C： water； D： methanol.

**表 2:** OPEs代谢物的质谱参数

Compound	ESI mode	Precursor ion （*m/z*）	Product ion （*m/z*）	Collision energy/V
Bis（1，3-dichloro-2-propyl） phosphate （BDCIPP）	‒	319	37	5
		317	35	5
1-Hydroxy-2-propyl bis（1-chloro-2-propyl） phosphate （BCIPHIPP）	‒	309	175	4
		309	99	17
Tris（2-chloroethyl） phosphate （TCEP）	+	285	99	21
		285	63	29
Dibutyl phosphate （DNBP）	‒	209	153	9
		209	79	20
4-Hydroxyphenyl-phenyl phosphate （4-HO-DPHP）	‒	265	108	50
		265	93	30
Diphenyl phosphate （DPHP）	‒	249	155	15
		249	93	30
Di-*o*-cresyl phosphate （DoCP）/di-*p*-cresyl phosphate （DpCP）	‒	277	107	28
		277	79	36
Bis（2-butoxyethyl） phosphate （BBOEP）	‒	297	197	10
		297	79	25
Bis（1-chloro-2-propyl） phosphate （BCIPP）	‒	251	35	5
		249	35	5
3-Hydroxyphenyl-diphenyl phosphate （3-HO-TPHP）	‒	341	249	17
		341	93	37
4-Hydroxyphenyl-diphenyl phosphate （4-HO-TPHP）	‒	341	249	17
		341	93	37
Bis（2-butoxyethyl） 3′-hydroxy-2-butoxyethyl phosphate （HO-TBOEP）	+	415	99	33
		415	45	25
2-Ethyl-5-hydroxyhexyl diphenyl phosphate （5-HO-EHDPP）	+	379	251	5
		379	153	30
2-Hydroxyethyl bis（2-butoxyethyl） phosphate （BBOEHEP）	+	343	243	4
		343	45	17
2-Ethylhexyl phenyl phosphate （EHPHP）	‒	285	93	33
		285	79	40

### 1.6 健康风险评估

使用尿液中OPEs代谢物的含量水平计算OPEs的每日摄入量（EDI，ng/（kg⋅d） bw），具体计算公式如下：


$$EDI=\frac{C\times V}{F\times bw}\times \frac{MW_{p}}{MW_{m}}$$ （1）

其中，*C*（ng/mL）是尿液中OPEs代谢物的质量浓度；*V*（mL）表示每日的尿排量（参考值：男性1 400 mL，女性1 200 mL）；*F*表示OPEs代谢物相对于母体化合物的尿排泄摩尔分数，DNBP和BBOEP为0.18，DPHP、4-HO-DPHP和BDCIPP为0.63；bw是体重（kg）；MW_p_和MW_m_（g/mol）是母体化合物以及对应代谢产物的摩尔质量^［22，23］^，其中4种主要单体磷酸三苯酯（TPHP）、磷酸三正丁酯（TNBP）、磷酸三丁氧乙酯（TBOEP）和磷酸三（1.3-二氯-2-丙基）酯（TDCIPP）的MW_p_分别为327、267、399和431 g/mol；其对应代谢产物4-HO-DPHP、DPHP、DNBP、BBOEP和BDCIPP的MW_m_分别为265、249、209、297和317 g/mol。此外，本研究进一步通过各个OPEs单体的非致癌风险参考剂量值（RfD）评估了主要单体的非致癌暴露风险。非致癌风险是指暴露于某种污染因子的敏感人群受到除癌症以外其他不良效应的可能性，其风险的大小用危害指数（HI）来表示，通过各种可能暴露途径和其相应的参考剂量进行确定。RfD为污染物在某种暴露途径下的口服参考剂量（ng/（kg⋅d） bw），表示在单位时间、单位体重摄取的不会引起人体不良反应的污染物的最大量。4种主要单体TPHP、TNBP、TBOEP和TDCIPP的RfD值分别为7 000、2 400、1 500和1 500 ng/（kg⋅d） bw^［5］^，具体计算公式如下：


$$HQ=\frac{EDI}{RfD}$$ （2）

$$HI=\sum HQ_{i}$$ （3）

其中，HQ为风险熵，用于表示各个单体的非致癌健康风险，HI表示多个OPEs单体暴露的累积危险指数。若HI<1，OPEs的暴露不会产生明显的非致癌健康风险；如果HI>1，则表明存在相关的非致癌健康风险^［24］^。尽管有报道指出OPEs具有致癌性^［4，25］^，但目前尚缺乏相应OPEs的致癌斜率因子，故而本文主要考虑OPEs的非致癌健康风险。

### 1.7 统计分析

本研究对检出率大于60%的OPEs代谢物进行统计分析，低于检出限（LOD）的浓度以LOD/ $\sqrt[{}]{2}$ 计。所有尿液中OPEs代谢物的水平使用肌酐进行校正。由于化学物的浓度呈偏态分布，因此将其经自然对数转换后纳入多元线性回归模型进行分析，并采用SPSS 25进行统计分析，双侧检验水准*α*=0.05。

## 2 结果与讨论

### 2.1 人口学特征

1 869名研究人员的平均年龄为（44.6±10.5）岁，平均BMI为（24.4±3.29） kg/m^2^，其中男性1 203人（64.4%），受教育水平至少为大学学历的有1 059人（56.7%），1 006人的月收入超过10 000元（53.8%），从不吸烟者的占比为69.5%，并且64.4%的参与者具有规律性的体育锻炼习惯。

### 2.2 尿液中OPEs代谢物的含量水平

本研究对尿液中15种OPEs代谢物进行了检测，其中6种OPEs代谢物的检出率超过60%，范围为62.2%~96.9%，各主要单体的详细测定结果见表3。总体而言，BBOEP的中位含量最高，达到0.56 ng/mL，高于挪威儿童和美国成年人尿液中的含量水平^［26‒28］^，这意味着相比于其他国家，我国居民可能更多地暴露于TBOEP。与BBOEP不同，DPHP和BDCIPP在本研究中的含量水平远低于美国、加拿大和澳大利亚等发达国家^［27，29，30］^。尽管BCIPHIPP在本研究中的中位含量水平高于其他OPEs代谢物（0.36 ng/mL），但相较于美国和澳大利亚的成人仍处于一个相对较低的含量水平^［31，32］^。目前，世界范围内关于尿液中4-HO-DPHP和DoCP/DpCP含量的研究报道仍十分有限，本研究中4-HO-DPHP的含量水平与比利时成年人尿液中的含量水平相当^［33］^，而DoCP/DpCP的含量水平则高于加拿大的普通人群^［34］^。上述结果表明，OPEs代谢物在人体尿液中的含量分布存在明显地区差异，这可能是由于OPEs主要用作阻燃剂，而不同国家防火标准之间的差异导致其用量不同进而影响了其代谢物在人体尿液中的含量水平^［4］^。因此，仍需开展广泛的内暴露监测以进一步阐明我国的OPEs人群内暴露特征。

**表 3:** 城市居民尿液中OPEs代谢物含量分布

Compound	LOD/（ng/mL）	Detection rate/%	Levels of OPE metabolites in urine/（ng/mL）					
			Geometric mean	Percentile				
				P5	P25	P50	P75	P95
BDCIPP	0.06	60.7	0.12	<LOD	<LOD	0.10	0.23	0.81
BCIPHIPP	0.10	92.8	0.35	<LOD	0.21	0.36	0.58	1.23
TCEP	0.09	55.9	0.15	<LOD	<LOD	0.12	0.32	0.94
DNBP	0.06	22.5	<LOD	<LOD	<LOD	<LOD	<LOD	1.32
4-HO-DPHP	0.04	62.2	0.15	<LOD	<LOD	0.12	0.42	3.50
DPHP	0.04	88.0	0.11	<LOD	0.06	0.11	0.19	0.45
DoCP/DpCP	0.03	90.6	0.10	<LOD	0.06	0.09	0.16	0.43
BBOEP	0.01	96.9	0.46	0.03	0.22	0.56	1.22	3.11

### 2.3 尿液中OPEs代谢物含量的影响因素分析

将尿液中OPEs代谢物的含量水平作为因变量，研究人群的性别、年龄、BMI、运动频率、是否吸烟、家庭收入以及各类食品的每周摄入频率作为自变量，使用多元线性回归模型，对影响尿液中OPEs代谢物含量水平的影响因素进行识别。如表4所示，女性尿液中BBOEP（标准化回归系数（*β*）=-0.238，95%置信区间（CI）：-0.362~-0.114）、BDCIPP（*β*=-0.104，95% CI：-0.162~-0.046）和BCIPHIPP（*β*=-0.061，95% CI：-0.100~-0.021）的含量水平均低于男性。这可能是由于相比于女性，男性可能更多的接触含有OPEs的电子产品以及男性通常具有更高的皮脂分泌，导致其皮肤会从空气中吸收更多的OPEs^［35］^。此外，仅有4-HO-DPHP（*β*=0.561，95% CI：0.042~1.080）在女性尿液中的含量水平高于男性。这可能是由于其母体TPHP在多种化妆品中广泛应用，女性使用化妆品可能导致摄入更多的TPHP^［36］^，从而使女性尿液中4-HO-DPHP的含量升高。尿液中BCIPHIPP（*β*=-0.003，95% CI：-0.004~-0.001）和DPHP（*β*=-0.002，95% CI：-0.003~-0.001）的含量水平随年龄的增长而降低，这是由于年轻人使用电子设备的频率更高，比老年人更多的接触OPEs^［37］^。BCIPHIPP（*β*=0.014，95% CI：0.003~0.025）和DoCP/DpCP（*β*=0.013，95% CI：0.002~0.024）在尿液中的含量水平与家庭年收入成正比，这主要归因于高收入家庭居住环境中更多的室内饰品以及更高的装修水平，导致其居住环境中更高的OPEs赋存水平^［38，39］^，这表明在住房装修的过程中应当避免过度装修，以减少OPEs的暴露。较高的运动频率有效地降低了尿液中BDCIPP（*β*=-0.086，95% CI：-0.139~-0.032）和BCIPHIPP（*β*=-0.049，95% CI：-0.085~-0.012）的含量水平，这可能是由于较高的运动频率提高了相关代谢酶的活性^［40］^，从而加快了OPEs的代谢，表现出对于上述两种代谢物母体OPEs暴露的保护作用。此外，坚果类的摄入频率与尿液中4-HO-DPHP（*β*=-0.471，95% CI：-0.926~-0.015）的含量水平呈现负相关。本研究中未发现吸烟和其他食品的摄入对于OPEs代谢物尿液含量水平的影响，这表明，对于本研究所纳入的人群而言，吸烟与食品的摄入并非OPEs的主要暴露途径，未来的研究应当扩大调查的范围以确定OPEs暴露的主要途径。

**表 4:** 尿液中OPEs代谢物含量影响因素的多元线性回归分析结果

Influencing factor	*β* （95% CI）					
	BDCIPP	BCIPHIPP	4-HO-DPHP	DPHP	DoCP/DpCP	BBOEP
Gender						
Male	0	0	0	0	0	0
Female	**-0.104** **（-0.162， -0.046）**	**-0.061** **（-0.100， -0.021）**	**0.561** **（0.042， 1.080）**	-0.008 （-0.04， 0.025）	-0.021 （-0.06， 0.018）	**-0.238** **（-0.362， -0.114）**
Age （years）	0.001 （-0.002， 0.003）	**-0.003** **（-0.004， -0.001）**	0.014 （-0.008， 0.035）	**-0.002** **（-0.003， -0.001）**	-0.001 （-0.003， 0）	0.001 （-0.004， 0.006）
BMI （kg/m²）	0.002 （-0.006， 0.010）	**0.009** **（0.003， 0.014）**	0.008 （-0.06， 0.077）	-0.002 （-0.006， 0.003）	-0.004 （-0.009， 0.001）	0.001 （-0.015， 0.018）
Educational level	0.010 （-0.015， 0.036）	-0.007 （-0.024， 0.01）	0.088 （-0.136， 0.313）	-0.011 （-0.025， 0.003）	-0.009 （-0.026， 0.008）	0.028 （-0.026， 0.082）
Family income	-0.001 （-0.018， 0.015）	**0.014** **（0.003， 0.025）**	0.079 （-0.069， 0.227）	0.002 （-0.007， 0.011）	**0.013** **（0.002， 0.024）**	-0.009 （-0.044， 0.027）
Smoking habit						
No	0	0	0	0	0	0
Yes	-0.021 （-0.066， 0.024）	0.010 （-0.021， 0.041）	-0.198 （-0.602， 0.206）	-0.002 （-0.027， 0.024）	0.007 （-0.023， 0.038）	-0.082 （-0.178， 0.014）
Exercise frequency （times/week）	**-0.086** **（-0.139， -0.032）**	**-0.049** **（-0.085， -0.012）**	-0.379 （-0.854， 0.097）	0.015 （-0.015， 0.044）	0.022 （-0.014， 0.058）	0.013 （-0.100， 0.127）
Diet						
Meet （times/week）	-0.072 （-0.193， 0.048）	-0.054 （-0.136， 0.029）	0.071 （-1.002， 1.144）	-0.013 （-0.08， 0.054）	0.030 （-0.051， 0.111）	0.033 （-0.223， 0.289）
Poultry （times/week）	-0.003 （-0.061， 0.054）	-0.014 （-0.053， 0.025）	0.284 （-0.23， 0.797）	0.006 （-0.026， 0.038）	0.009 （-0.03， 0.048）	-0.035 （-0.158， 0.088）
Vegetable （times/week）	0.038 （-0.202， 0.277）	0.152 （-0.011， 0.316）	0.869 （-1.261， 2.999）	-0.011 （-0.144， 0.122）	-0.114 （-0.275， 0.047）	-0.319 （-0.827， 0.19）
Egg （times/week）	-0.070 （-0.164， 0.024）	0.013 （-0.05， 0.077）	-0.223 （-1.053， 0.606）	-0.015 （-0.067， 0.037）	0.026 （-0.036， 0.089）	0.126 （-0.072， 0.324）
Fruit （times/week）	-0.026 （-0.1， 0.047）	-0.051 （-0.102， -0.001）	-0.140 （-0.796， 0.516）	-0.006 （-0.047， 0.035）	0.015 （-0.034， 0.065）	-0.012 （-0.168， 0.145）
Milk （times/week）	-0.033 （-0.088， 0.022）	0.036 （-0.002， 0.073）	0.337 （-0.154， 0.828）	0.009 （-0.022， 0.039）	0.031 （-0.006， 0.068）	0.056 （-0.061， 0.173）
Nut （times/week）	0.014 （-0.037， 0.065）	-0.016 （-0.051， 0.019）	**-0.471** **（-0.926， -0.015）**	0.006 （-0.023， 0.034）	-0.017 （-0.051， 0.017）	-0.053 （-0.162， 0.055）
Pickle （times/week）	0.019 （-0.028， 0.067）	0.011 （-0.021， 0.044）	-0.116 （-0.536， 0.304）	-0.002 （-0.028， 0.024）	0.024 （-0.008， 0.056）	0.110 （0.009， 0.210）

95% CI： 95% confidence intervals. Values in bold indicated statistical significance （*P*<0.05）.

### 2.4 OPEs暴露评估

#### 2.4.1 OPEs暴露水平

由于缺乏全面的暴露计算参数，本研究对可获得参数的4种OPEs进行了暴露水平评估，包括TDCIPP、TNBP、TPHP和TBOEP。本研究中所有OPEs的整体EDI值为5.60~2 800 ng/（kg⋅d） bw，其中位值为104 ng/（kg⋅d） bw。在4种OPEs中，TBOEP的EDI值最高，其中位值为57.2 ng/（kg⋅d） bw（1.11~1 330 ng/（kg⋅d） bw），其对OPEs总EDI值的贡献高达55.6%（图1）。此外，TNBP的暴露水平同样不容忽视，其中位EDI值为32.4 ng/（kg⋅d） bw（0.138~2 000 ng/（kg⋅d） bw），占OPEs总EDI值的31.5%。性别差异分析显示，男性的OPEs暴露水平高于女性，男性中位EDI值为112 ng/（kg⋅d） bw（6.03~2 670 ng/（kg⋅d） bw），与之相比，女性的中位EDI值仅为89.9 ng/（kg⋅d） bw（5.61~2 800 ng/（kg⋅d） bw）。对于不同性别而言，TBOEP均为最主要的暴露单体，其对于男性和女性整体EDI值的贡献分别为57.2%（62.8 ng/（kg⋅d） bw，1.11~1 330 ng/（kg⋅d） bw）和53.8%（52.1 ng/（kg⋅d） bw，1.13~1 250 ng/（kg⋅d） bw）。上述结果表明，TBOEP可能是人体最主要的暴露物质，这可能是由于TBOEP不仅用作阻燃剂，还被用作增塑剂而广泛添加在各类室内装修材料中，进而导致其通常在室内环境中具有更高的含量水平^［41，42］^，提示未来研究应当更加注重对于环境中TBOEP的监测。虽然本研究的人群涵盖了较宽的年龄跨度（18~89岁），但不同年龄段人群OPEs的暴露水平未见显著差异。

**图1:**
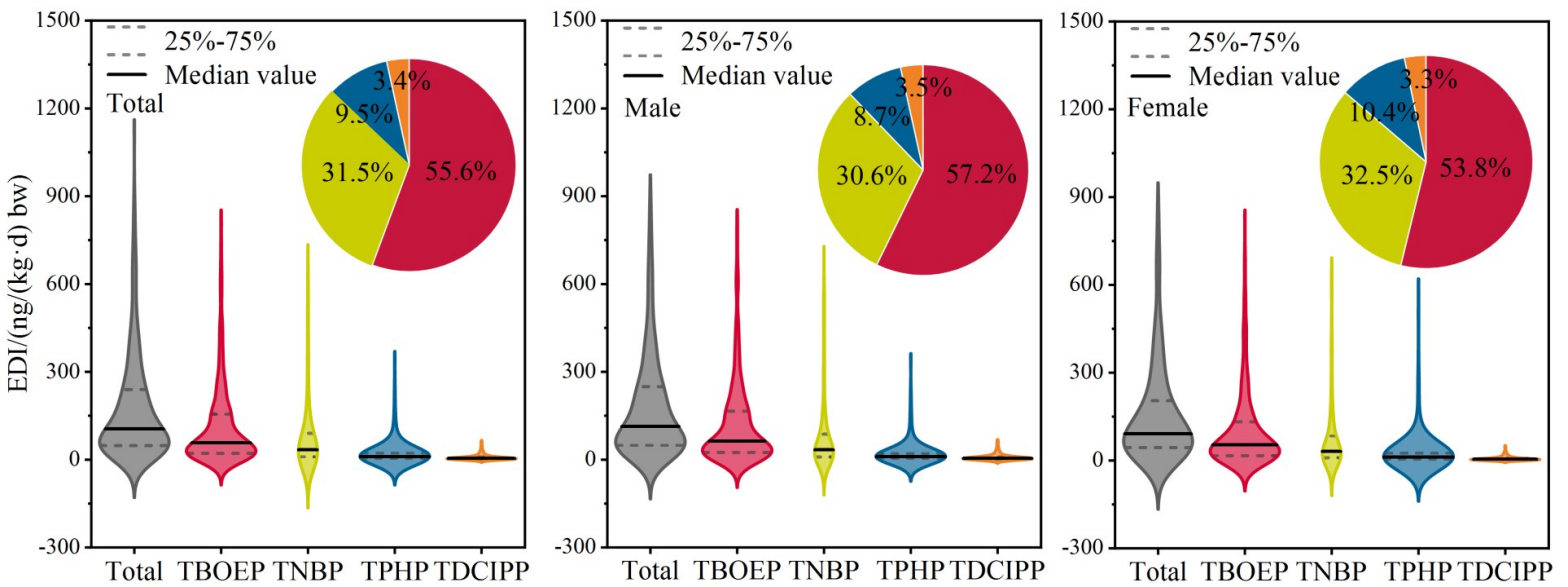
OPEs的每日摄入量

#### 2.4.2 OPEs健康风险

在本研究中OPEs的HI值范围为0.000 2~1.03，对应中位HI值为0.06。4种OPEs的暴露风险依次为TBOEP（中位值：0.038，范围：0.000 7~0.883）>TNBP（0.013，0.000 05~0.833）>TDCIPP（0.002，0.000 8~0.288）>TPHP（0.001，0.000 2~0.350），分别贡献了总HI值的68.9%、24.4%、4.2%和2.5%（图2）。在所有研究对象中，相较于女性（0.049，0.002~0.840），男性的HI值更高（0.061，0.002~1.03），且TBOPE均为主要高风险单体，分别贡献了总HI值的70.1%（0.042，0.000 7~0.883）和67.6%（0.035，0.000 8~0.835）（图2）。相较于其他单体，TBOEP对于健康风险的贡献普遍比暴露量更高，主要归因于TBOEP较高的毒性以及相对较低的健康阈值，这表明应当特别重视TBOEP暴露所带来的负面健康影响。尽管TDCIPP的暴露水平低于TPHP，但由于其更低的健康阈值，TDCIPP表现出更高的健康风险。在不同年龄段研究对象的健康风险之间未发现明显差异。在本研究所纳入的全部研究对象中，仅有1名男性的HI值大于1，这意味着对于绝大部分研究对象并未观察到明显的健康风险。然而，考虑到本研究纳入的OPE单体数量有限，且OPEs可能与其他污染物之间存在复合暴露效应，因此建议后续应对OPEs进行更全面的暴露检测并纳入更多类别的污染物进行复合健康风险的全面评估。

**图2:**
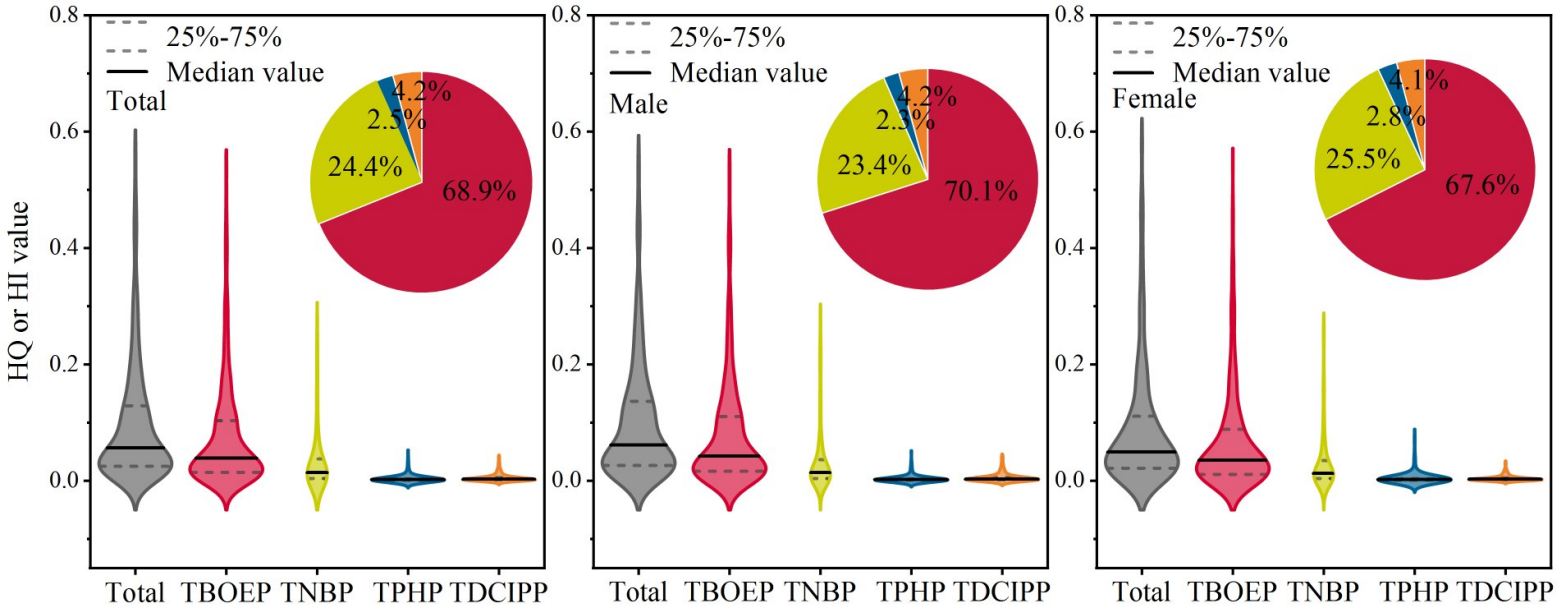
OPEs的HQ和HI

## 3 结论

本研究发现我国城市居民普通成人普遍暴露于多种OPEs，性别、年龄、家庭收入和运动频率是影响尿液中OPEs代谢物含量的因素。TBOEP和TNBP为高暴露单体，TBOEP为高风险单体。性别是影响暴露水平和健康风险的重要因素，男性普遍表现出更高的暴露水平和潜在健康风险。相比之下，年龄对于暴露水平和健康风险的影响并不显著。此外，吸烟和肉类摄入并非OPEs的主要暴露路径。本研究阐明了人体尿液中OPEs代谢物含量水平及其特征，评估了OPEs的暴露水平以及相应的健康风险。结果显示，绝大部分人群不存在明显的健康风险，但在未来应进一步扩大污染物人体生物监测的范围，深入探讨化学污染物，特别是多污染物复合暴露对人体健康的影响。
